# Supplementary material for: Near-chromosome level genome assembly of the fruit pest Drosophila suzukii using long-read sequencing
Source: Sci Rep. 2020 Jul 8;10:11227. doi: 10.1038/s41598-020-67373-z (PMC7343843; doi:10.1038/s41598-020-67373-z)
Supplement: Supplementary file 1 — Supplementary file1 (PDF 1614 kb) [file 41598_2020_67373_MOESM1_ESM.pdf]

Supplementary Figures

Near-chromosome level genome assembly of the fruit pest *Drosophila suzukii* using long-read sequencing

Mathilde Paris[1,2], Roxane Boyer[1,3], Rita Jaenichen[4], Jochen Wolf[4,5], Marianthi Karageorgi[1,6], Jack Green[1], Mathilde Cagnon[4], Hugues Parinello[7], Arnaud Estoup[8], Mathieu Gautier[8,*], Nicolas Gompel[4,*], Benjamin Prud'homme[1,*]

[1] Aix-Marseille Université, CNRS, IBDM, Institut de Biologie du Développement de Marseille, 13288 Marseille Cedex 9, France

[2] present address: IGFL, Institut de Génomique Fonctionnelle de Lyon, Université de Lyon, Ecole Normale Supérieure de Lyon, CNRS, Université Claude Bernard Lyon 1, UMR 5242, 69364 Cedex 07, Lyon, France.

[3] present address: INRA, US 1426, GeT-PlaGe, Genotoul, Castanet-Tolosan, France

[4] Ludwig-Maximilians Universität München, Fakultät für Biologie, Biozentrum, Grosshaderner Strasse 2, 82152 Planegg-Martinsried, Germany

[5] Department of Ecology and Genetics, Evolutionary Biology Centre, Uppsala University, Norbyvägen 14-18, 75236, Uppsala, Sweden

[6] present address: Department of Integrative Biology, University of California, Berkeley, CA, USA

[7] MGX, MGX, Biocampus Montpellier, CNRS, INSERM, Univ Montpellier, Montpellier, France

[8] CBGP, INRA, CIRAD, IRD, Montpellier SupAgro, Univ Montpellier, Montpellier, France

[*] co-corresponding authors

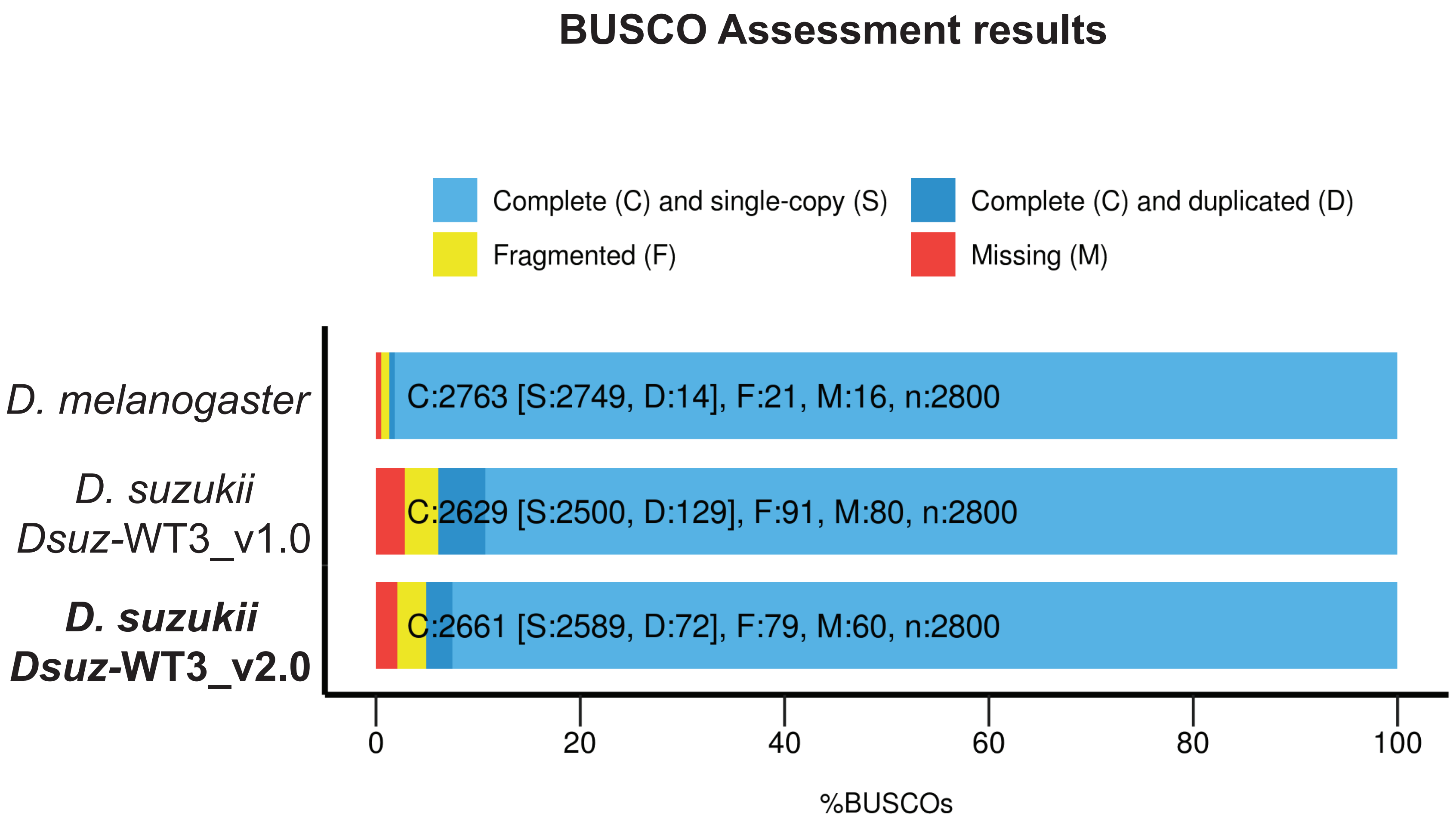
BUSCO Assessment results
Complete (C) and single-copy (S)
Complete (C) and duplicated (D)
Fragmented (F)
Missing (M)
D. melanogaster
D. suzukii Dsuz-WT3_v1.0
D. suzukii Dsuz-WT3_v2.0
C:2763 [S:2749, D:14], F:21, M:16, n:2800
C:2629 [S:2500, D:129], F:91, M:80, n:2800
C:2661 [S:2589, D:72], F:79, M:60, n:2800
0
20
40
60
80
100
%BUSCOs

Figure S1

Figure S1: BUSCO statistics for the different assembly steps.

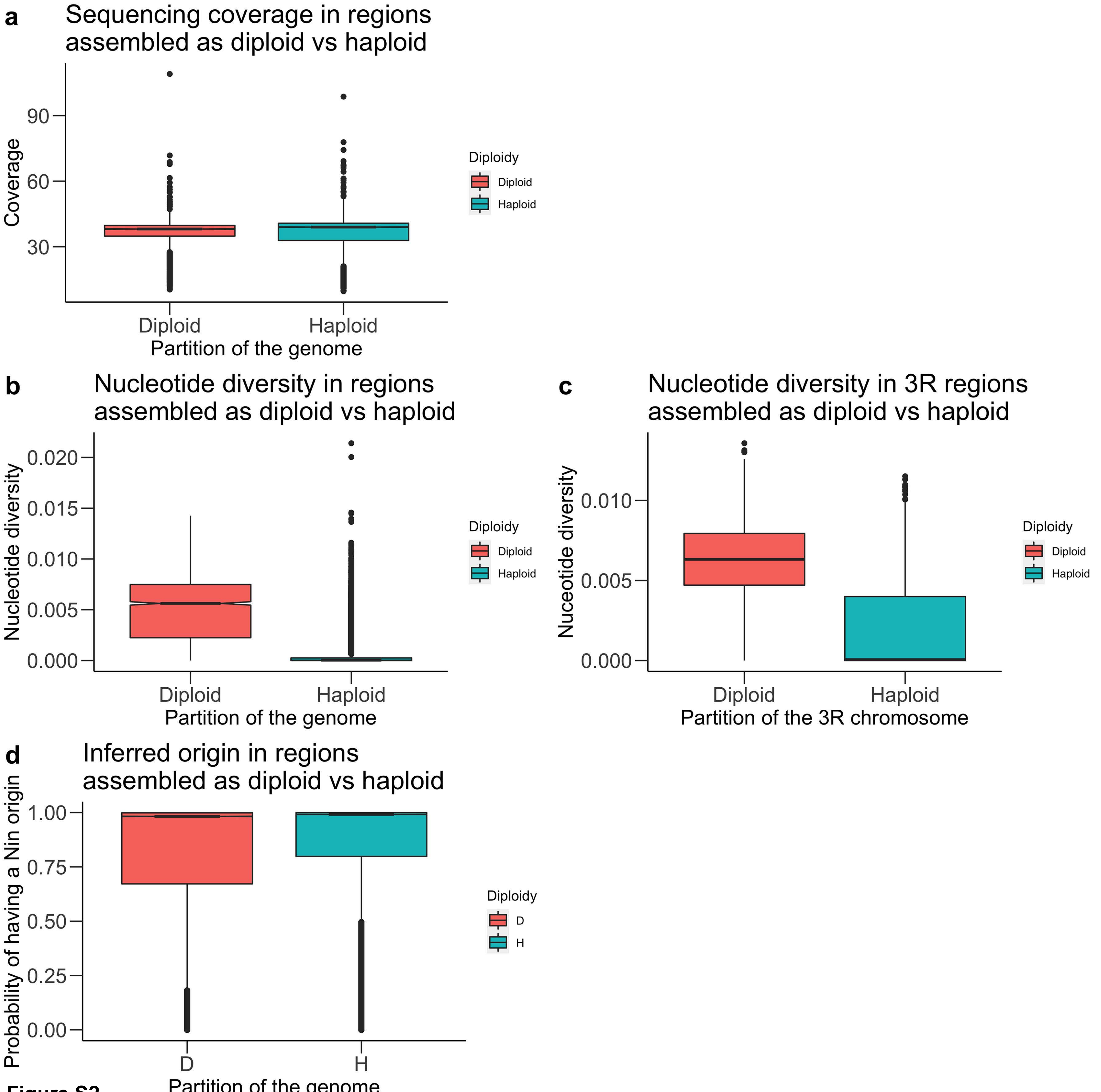
a
Sequencing coverage in regions assembled as diploid vs haploid
Coverage
90
60
30
Diploid
Haploid
Partition of the genome
Diploidy
Diploid
Haploid
b
Nucleotide diversity in regions assembled as diploid vs haploid
Nucleotide diversity
0.020
0.015
0.010
0.005
0.000
Diploid
Haploid
Partition of the genome
Diploidy
Diploid
Haploid
c
Nucleotide diversity in 3R regions assembled as diploid vs haploid
Nuceotide diversity
0.010
0.005
0.000
Diploid
Haploid
Partition of the 3R chromosome
Diploidy
Diploid
Haploid
d
Inferred origin in regions assembled as diploid vs haploid
Probability of having a Nin origin
1.00
0.75
0.50
0.25
0.00
D
H
Partition of the genome
Diploidy
D
H

Figure S2

Figure S2 : Comparison of coverage (**A**) and nucleotide diversity (**B**) between regions for which an alternative sequence was assembled (« Diploid ») and the rest of the genome (« Haploid »). Reads from the Dsuz-WT3_v2.0 Pool-seq sample were used and mean coverage was calculated on 10 kb windows. (**C**) Same analysis than in (**B**) for the contigs associated with chromosomal arm 3 only. (**D**) Probability of being of population CN-NIN (Ningbo, China) origin for SNPs located within either the diploid or the haploid regions of the genome. A value close to 1 means that the SNP is predicted as being in a genomic region of CN-NIN origin and a value close to 0 means that the SNP is predicted as being in a region of alternative populational origin (*i.e.*, US-Haw for Hawaii – USA).

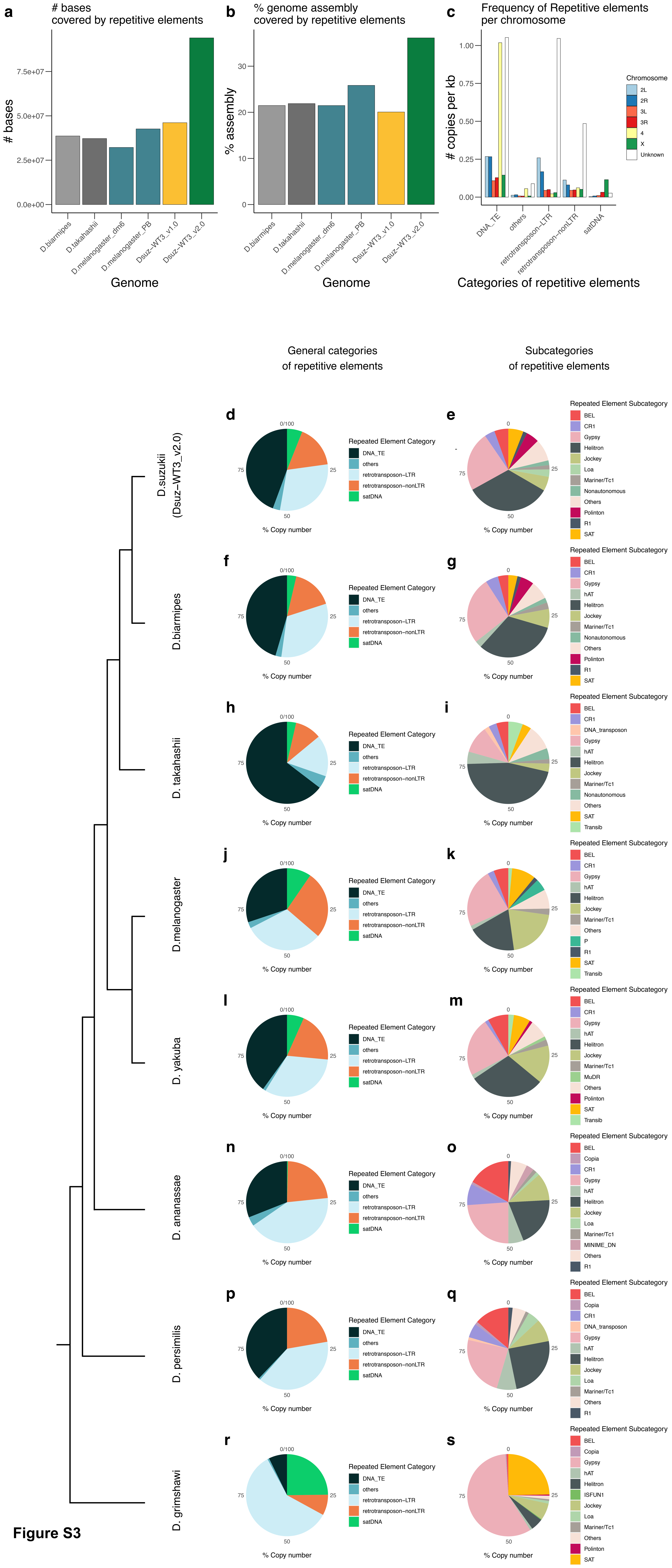
a
bases
covered by repetitive elements
bases
0.0e+00
2.5e+07
5.0e+07
7.5e+07
D.biarmipes
D.takahashii
D.melanogaster_dm6
D.melanogaster_PB
Dsuz−WT3_v1.0
Dsuz−WT3_v2.0
Genome
b
% genome assembly
covered by repetitive elements
% assembly
0
10
20
30
Genome
c
Frequency of Repetitive elements
per chromosome
copies per kb
0.00
0.25
0.50
0.75
1.00
Chromosome
2L
2R
3L
3R
4
X
Unknown
DNA_TE
others
retrotransposon−LTR
retrotransposon−nonLTR
satDNA
Categories of repetitive elements
General categories
of repetitive elements
Subcategories
of repetitive elements
D.suzukii
(Dsuz−WT3_v2.0)
D.biarmipes
D. takahashii
D.melanogaster
D. yakuba
D. ananassae
D. persimilis
D. grimshawi
d
e
f
g
h
i
j
k
l
m
n
o
p
q
r
s
0/100
25
50
75
0
% Copy number
Repeated Element Category
DNA_TE
others
retrotransposon−LTR
retrotransposon−nonLTR
satDNA
Repeated Element Subcategory
BEL
Copia
CR1
DNA_transposon
Gypsy
hAT
Helitron
ISFUN1
Jockey
Loa
Mariner/Tc1
MINIME_DN
MuDR
Nonautonomous
Others
P
Polinton
R1
SAT
Transib

Figure S3

Figure S3: Comparison of the repetitive sequences repertoire between *D suzukii*, and other *Drosophila* species. Comparison of (**A**) the number of bases and (**B**) the percentage of the genome assembly covered by repetitive elements in *D. suzukii*, *D. melanogaster*, *D. biarmipes* and *D. takahashii.* Two assemblies were used for *D. suzukii* (Dsuz-WT3-v1.0 and Dsuz-WT3_v2.0) and for *D. melanogaster* (dm6 and an assembly done using PacBio (PB) reads), (**C**) Chromosomal distribution of broad categories of repetitive elements in Dsuz-WT3_v2.0 (**D-S**) The number of copies of each type of repetitive elements was compared in *D. suzukii*, *D. melanogaster*, *D. biarmipes*, *D. takahashii*, *D. yakuba*, D. *ananassae*, *D. persimilis* and *D. grimshawi* for broad categories (**D, F**, **H**, **J**, **L, N**, **P** and **R**) and more specific categories (**C**, **E**, **G**, **I**, **K**, **M**, **O**, **Q** and **S**) of repetitive elements. Species phylogeny is indicated.

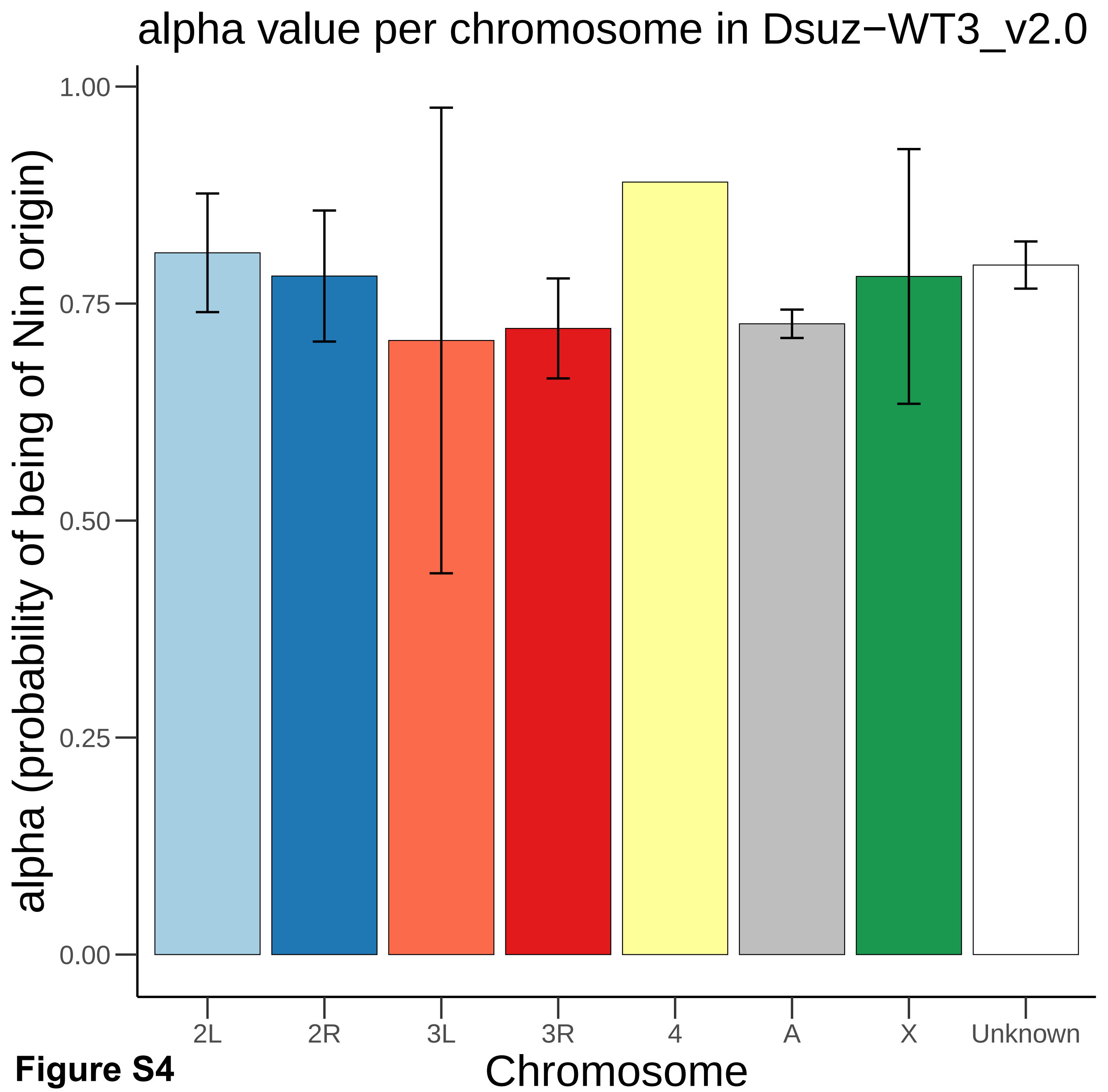
alpha value per chromosome in Dsuz−WT3_v2.0
alpha (probability of being of Nin origin)
1.00
0.75
0.50
0.25
0.00
2L
2R
3L
3R
4
A
X
Unknown
Chromosome

Figure S4

Figure S4: Chromosomal distribution of alpha, the probability of being of population CN-NIN (Ningbo, China). Values correspond to the relative length proportion of each contig with a Nin origin. “2L”, “2R”, “3L”, “3R”, “4”, “X”: assigned *D. melanogaster* chromosome for each Dsuz-WT3_v2.0 contigs. “A”: autosomal contigs with no clear corresponding *D. melanogaster* chromosome. “Unknown”: contigs for which chromosomal features (i.e., assignment to autosomal or X chromosomes and to *D. melanogaster* chromosome arm) remain unknown. Error bars correspond to S.E.M.

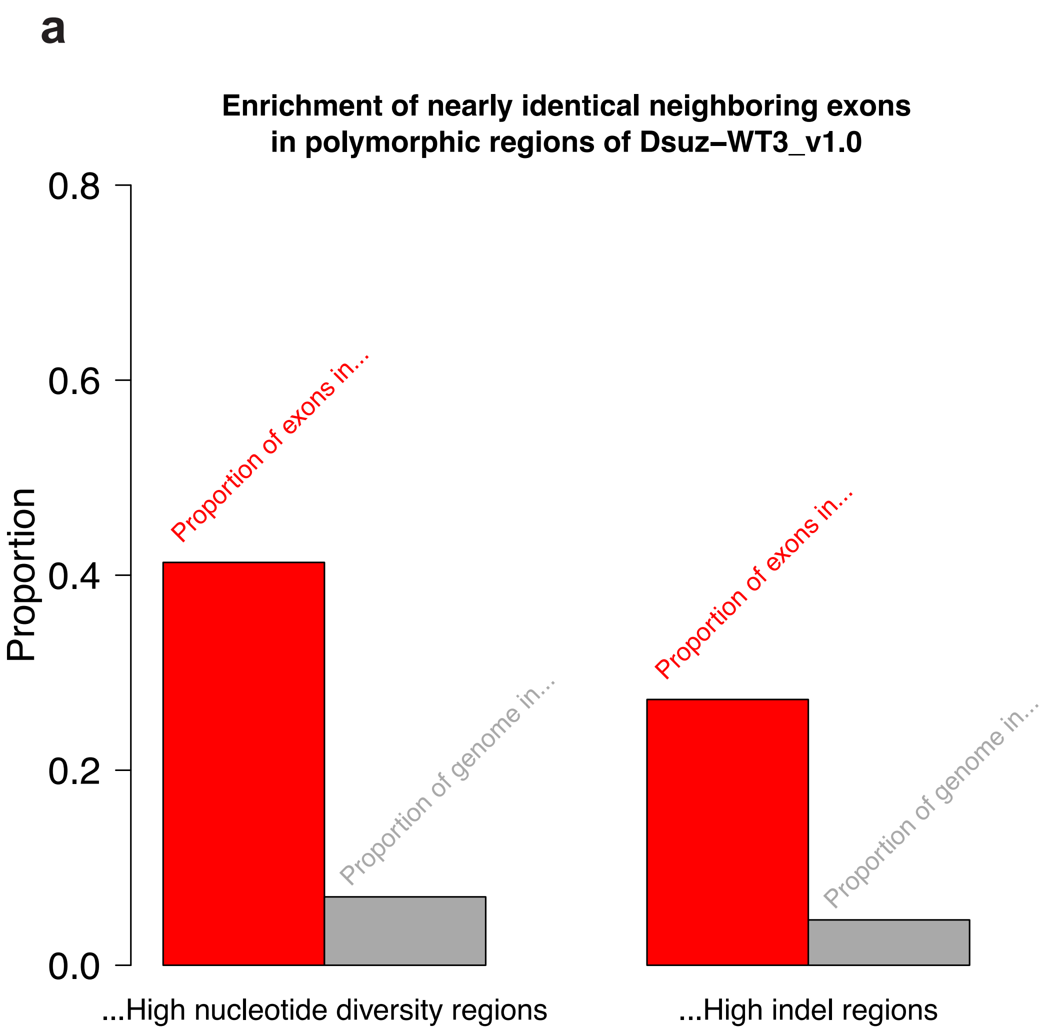
a
Enrichment of nearly identical neighboring exons
in polymorphic regions of Dsuz–WT3_v1.0
Proportion
0.8
0.6
0.4
0.2
0.0
Proportion of exons in...
Proportion of genome in...
Proportion of exons in...
Proportion of genome in...
...High nucleotide diversity regions
...High indel regions

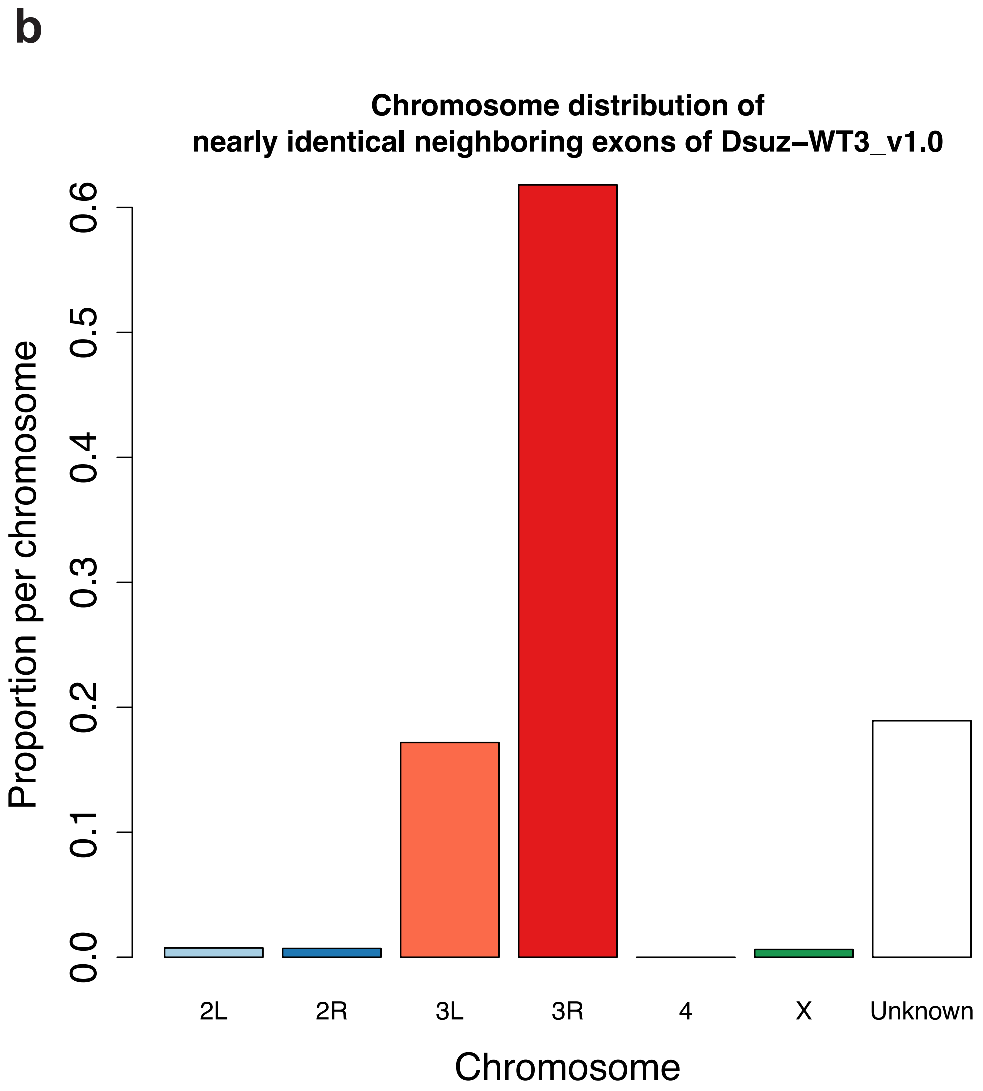
b
Chromosome distribution of
nearly identical neighboring exons of Dsuz–WT3_v1.0
Proportion per chromosome
0.6
0.5
0.4
0.3
0.2
0.1
0.0
2L
2R
3L
3R
4
X
Unknown
Chromosome

Figure S5

Figure S5: Nearly identical neighboring exons of Dsuz-WT3_v1.0 map to Dsuz-WT3_v2.0 regions of high nucleotide diversity and to *D. melanogaster* chromosomal arms 3R and 3L. We aligned the Dsuz-WT3_v1.0 assembly to the Dsuz-WT3_v2.0 assembly and dm6 and determined the location of the nearly identical neighboring exons of Dsuz-WT3_v1.0 respective to the regions of Dsuz-WT3_v2.0 high nucleotide diversity as a proxy (underestimate) of nucleotide diversity in Dsuz-WT3_v1.0. (**a**) In red, Proportion of the nearly identical neighboring exons in regions of high nucleotide diversity (~19Mb) or high indel rates (~12Mb). For comparison, the proportion of those regions in the Dsuz-WT3_v2.0 assembly is indicated in grey. Those exons are *ca.* 6 fold more abundant than expected, Fisher's test p.value < $2.2x10^{-16}$. (**b**) Alignment of the Dsuz-WT3_v1.0 nearly identical neighboring exons on *D. melanogaster* chromosomes. "2L", "2R", "3L", "3R", "4", "X": assigned *D. melanogaster* chromosome for each Dsuz-WT3_v1.0 contigs. "Unknown": contigs for which chromosomal features (i.e., assignment to *D. melanogaster* chromosome arm) remain unknown.

Table S1: Descriptive statistics of the *D. suzukii* assemblies Dsuz-WT3_v2.0 and Dsuz-WT3_v1.0, and of the *D. melanogaster* assembly dm6.

Table S2: Gene orthology table based on genome alignment between Dsuz-WT3_v2.0, Dsuz-WT3_v1.0 (Chiu et al., 2013) and *D. melanogaster* r6.03.

Table S3: Chromosome association and summary statistics per Contig.

Chromosome association was based on (i) sequence similarity + syntheny with the *D. melanogaster* genome assembly and (ii) sex ratio of reads issued from a female or a male. 2R, 2L, 3R, 3L, 4, X are chromosome names. "A": Autosome. NA: no assignation could be made. Contigs with no estimation of alpha did not have enough SNPs associated. alpha represents an estimation of the mean fraction of the assembled genome with a Ningbo origin. rho is a parameter of the HMM model and could be described as "recombination" rate between ancestral populations. See Supplementary File S2 for further details.
