# Supplementary material for: Near-chromosome level genome assembly of the fruit pest Drosophila suzukii using long-read sequencing
Source: Sci Rep. 2020 Jul 8;10:11227. doi: 10.1038/s41598-020-67373-z (PMC7343843; doi:10.1038/s41598-020-67373-z)
Supplement: Supplementary file 4 — Supplementary file4 (PDF 49 kb) [file 41598_2020_67373_MOESM4_ESM.pdf]

Table S1*:* Descriptive statistics of the *D. suzukii* assemblies Dsuz-WT3_v2.0 and Dusz-WT3_v1.0, and of the *D. melanogaster* assembly dm6.

	***D. suzukii* Dsuz-WT3_v2.0**	***D. suzukii* Dsuz-WT3_v1.0**	***D. melanogaster dm6***
**# contigs**	546	4813	1870
**Largest contig**	25 589 241	22 559 587	32 079 331
**Total length**	268 012 156	231 788 855	143 726 002
**N50**	2 609 782	397 157	25 286 936
**L50**	15	73	3
